# Social/economic costs and health-related quality of life in patients with spinal muscular atrophy (SMA) in Spain

**DOI:** 10.1186/s13023-017-0695-0

**Published:** 2017-08-18

**Authors:** Julio López-Bastida, Luz María Peña-Longobardo, Isaac Aranda-Reneo, Eduardo Tizzano, Mark Sefton, Juan Oliva-Moreno

**Affiliations:** 1 Faculty of Occupational Therapy, Speech Therapy and Nursing, University of Castilla-La Mancha, Talavera de la Reina. Toledo, Spain; 2 Faculty of Law and Social Sciences, University of Castilla-La Mancha, Toledo, Spain; 3 Department of Clinical and Molecular Genetics and CIBERER, Hospital Vall d’Hebron, Barcelona, Spain; 4 BiomedRed, Madrid, Spain

**Keywords:** Spinal muscular atrophy, Cost-of-illness, Health-related quality of life, Economic burden, Spain

## Abstract

**Background:**

The aim of this study was to determine the economic burden and health-related quality of life (HRQOL) of patients with Spinal Muscular Atrophy (SMA) and their caregivers in Spain.

**Methods:**

This was a cross-sectional and retrospective study of patients diagnosed with SMA in Spain. We adopted a bottom up, prevalence approach design to study patients with SMA. The patient’s caregivers completed an anonymous questionnaire regarding their socio-demographic characteristics, use of healthcare services and non-healthcare services. Costs were estimated from a societal perspective (including healthcare costs and non-healthcare costs), and health-related quality of life (HRQOL) was assessed using the EQ-5D questionnaire. The main caregivers also answered a questionnaire on their characteristics and on their HRQOL.

**Results:**

A total of 81 caregivers of patients with different subtypes of SMA completed the questionnaire. Based on the reference unitary prices for 2014, the average annual costs per patient were € 33,721. Direct healthcare costs were € 10,882 (representing around 32.3% of the total cost) and the direct non-healthcare costs were € 22,839 (67.7% of the total cost). The mean EQ-5D social tariff score for patients was 0.16, and the mean score of the EQ-5D visual analogue scale was 54. The mean EQ-5D social tariff score for caregivers was 0.49 and their mean score on the EQ-5D visual analogue scale was 69.

**Conclusion:**

The results highlight the burden that SMA has in terms of costs and decreased HRQOL, not only for patients but also for their caregivers. In particular, the substantial social/economic burden is mostly attributable to the high direct non-healthcare costs.

## Introduction

Spinal muscular atrophy (SMA) is an autosomal recessive neuromuscular disorder caused by the degeneration of alpha motor neurons in the anterior horns of the spinal cord. SMA is the second most common severe hereditary disease of infancy and early childhood, with an estimated incidence of 1/5000 to 1/10,000 births and a carrier frequency of 1/35 to 1/50 [1, 2].

The difficulties of living with SMA begin with the long and often arduous process of diagnosis. Therefore, as is the case with other rare diseases [3], SMA imposes a considerable economic burden on society. Substantial health-care costs are associated with its management in terms of treatment, hospitalizations, emergency consultations, visits to General Practitioners (GPs) and other specialists, etc. Additionally, due to the considerable disability that SMA causes, most of the patients are unable to fulfill their activities of daily living (ADLs) and they rely on family for support and/or social services. Therefore, quantification of the economic burden of SMA needs not only to consider the costs to the healthcare systems but also, the costs of formal care, unpaid care provided by relatives and other household costs.

To our knowledge, few studies have delved into the cost of SMA from a social point of view [4]. Therefore, the main aim of this study was two-fold. First, to estimate the economic costs related to SMA from a societal perspective in Spain Second, to assess the HRQOL of SMA patients and their caregivers. This information provides valuable information on the real economic impact that SMA has in Spain.

## Methods

### Participants and procedures

This was a cross-sectional and retrospective study of patients diagnosed with SMA who received outpatient care at the time of the study but who had also received inpatient care at some time. The Strengthening the Reporting of Observational Studies in Epidemiology (STROBE) guidelines have been followed in the study [5]. All the patients were living in Spain at the time of the study. The fieldwork was carried out between July 2015 and November 2015, and patient associations provided the careers with access to the online questionnaires by e-mail. Those eligible to participate in this study were children/adolescents diagnosed with SMA and their main caregiver, the latter completing the self-administered questionnaire to provide information on: public healthcare and non-healthcare resource use; the costs of professional private care; informal care required to manage SMA; their health related quality of life (HRQOL); and socio-demographic parameters. Study materials were presented for review by the local experts to ensure the questionnaire was comprehensible, accurate and complete. A pilot study was carried out between May 2015 and June 2015 to further establish the validity of the questionnaire.

The SMA patients were classified into three types based on age at disease onset and the clinical severity of the disease, as defined by the international SMA Consortium [6, 7]. Type I SMA is the most severe form, characterized by generalized muscle weakness and hypotonia at birth or within the first 6 months of live. These children are never able to sit unaided and less than 10% survive for 2 years without invasive respiratory support [8]. Type II SMA is distinguished by the age of onset, within the first 18 months of life. The disease is less aggressive and they survive longer than type I patients, mainly in function of the appearance of severe respiratory, orthopedic and nutritional complications. Type III SMA is the milder form of SMA in which weakness develops in patients who were previously able to sit and walk unassisted [9]. Due to the small overall sample size, it was not possible to carry out an estimate of cost separately for each SMA disease type. However, due to the importance of type II in such disease, it has been included information about HRQOL as well as costs.

All caregivers were informed about the study objectives and the confidentiality of the data, and they were asked to confirm their understanding of the study conditions when they agreed to participate. The survey was completely anonymously as the participants were contacted by their patient organizations and their responses were sent directly to the researchers without including any personal information (such as name, identification, address, or e-mail).

### Costing methodology

A prevalence approach was used to estimate costs from a societal perspective. Disease prevalence takes into account all existing cases during a given year and all healthcare resources used for prevention, treatment and rehabilitation, as well as the allocation of other resources (formal and informal care) as a consequence of the illness [10–13]. To estimate resource utilization, the questionnaire solicited information covering the 6 month period prior to the study (12 months for hospitalizations). The questionnaire was detailed enough to reduce both exaggeration and underestimation. The resources used were multiplied by the unit costs to estimate the annual cost per patient using 2014 as the reference year.

### Direct healthcare costs

Information about the number of hospital admissions, drug consumption, emergency visits, outpatient care (rehabilitation, medical tests, medical visits and home medical care), healthcare-related transportation and the use of devices was obtained from the questionnaires. When the useful life of a device exceeds the period of analysis, an amortization period of 4 years was considered. The cost of drugs used by patients was calculated by determining the daily cost for each of the products used (based on the cost of each pack dispensed and the dose used), multiplying by the duration of use. We applied local reference prices [14–17] to the healthcare resource data (drugs, healthcare, clinical tests, hospitalization and health supplies) to estimate the health cost of the illness.

### Direct non–healthcare costs

We also obtained data on formal (social services) and informal care. Informal care was defined as the tasks performed by non-professionals that help maintain or enhance a patient’s autonomy. Therefore, informal services are mainly provided by relatives. Information about informal care was obtained from the questionnaires, specifically from the items concerning the time spent helping the patient with his or her basic ADLs and the time spent helping with necessary instrumental ADLs (recall method). As a conservative criterion, and to prevent joint production bias, we censored the time of care to a maximum of 16 h per day per caregiver (112 h per week) when the reported time exceeded this figure. The approach used to evaluate the care hours was the proxy good method, which values time as an output [18–20]. This method evaluates the care provided by the informal caregiver, considering that if he/she did not provide these services, they would have to be substituted by another person who could provide them. Therefore, we took into consideration how much said substitution would cost or the cost of hiring a professional caregiver to perform these tasks [15]. Hence, informal caregiving hours were valued using a professional care wage in Spain in 2014 of 13.24€ per hour.

Formal care refers to the community care and home care provided by professionals. It may be publicly or privately funded, or mixed. Information on formal paid care provided by professional caregivers and other social services was obtained from the questionnaires under the category of social services and the unit costs used to value formal services were obtained from public Spanish sources [15]. In the case of other non-healthcare family costs, we applied the same period of amortization used for medical devices to housing and vehicle adaptations (4 years).

### Patient and caregiver outcomes

Patient and caregiver outcomes were obtained by means of proxy or self-administered questionnaires, such as the EQ-5D-3 L proxy for patients and the EQ-5D-5 L for caregivers, or the Barthel Index and the Zarit burden interview. These questionnaires have been used in other similar studies of patients with rare diseases [21]. The EQ-5D is a simple generic instrument developed by a multidisciplinary group of researchers [22] and it has been validated in many European countries. It is a commonly used tool in economic evaluation and health technology assessment [23]. HRQOL is defined in terms of five dimensions: mobility, self-care, everyday activities, pain/discomfort and anxiety/depression [24]. The values or utilities are indicated on a scale where 0 corresponds to death and 1 corresponds to perfect health, with negative values also being possible. The second part of the EQ-5D consists of a vertical 20-cm, 0-100 visual analogue scale (VAS), where 0 represents the worst imaginable health state and 100 represents the best imaginable health state. The respondent marks a point on the scale to reflect their overall health on the day of the interview [24].

The EQ-5D-3 L proxy, which has only been validated for adult responders, was used as a proxy-reported HRQOL of all patients (1-17 years), the responses being provided by their primary caregivers. However, some studies have used this scale in children [25, 26] and parent-proxy ratings have been shown to be both feasible and valid [27, 28].

The Barthel Index is a widely used tool to assess disability and it measures the ability of a person to perform 10 basic ADLs, providing a quantitative estimate of the subject’s degree of dependence [29–31]. The Barthel Index is recommended to measure physical disability both in clinical practice and public health research [30].

Caregivers also completed the Zarit burden interview (22-item version), which measures the subjective burden among caregivers. Each item is a statement to which the caregiver is asked to respond on a 5-point scale, with options ranging from 0 (never) to 4 (nearly always) [32]. The total score ranges from 0 to 88, with scores under 21 corresponding to little or no burden and scores above 61 to severe burden.

### Statistical methods

Descriptive analysis was carried out using mean and standard deviation (SD) in continuous variables and proportions for dichotomous or categorical variables. All analysis was carried out with Stata SE (v 14.2).

## Results

A total of 95 completed questionnaires were collected related to individuals with SMA, of which 14 were excluded because the patients did not fulfill the inclusion criteria. Thus, a total valid sample of 81 patients was studied. The main characteristics of the participants and their caregivers are shown in Table 1. The average age of the children was 7.22 years (SD = 5.47) and 42% were boys. In this sample, 10% of patients had type I SMA, 74% of patients had SMA type II and 16% type III SMA. It is noteworthy that it seems to take a long time to obtain a diagnosis for this condition, perhaps because the average age of the first symptom is 4.32 years (SD = 2.5).

**Table 1:** Demographic characteristic of the study participants (*N* = 81 patient-caregiver pairs)

	n (%)
No. of Patients (%)	81 (100)
Type I	8 (9.87)
Type II	60 (74.07)
Type III	13 (16.06)
Gender	
Female	47 (58.02)
Male	34 (41.98)
Age (mean)	7.22 (5.47)^a^
Age from the first symptom, mean (SD)	4.32 (2.50)
Type I	1.55 (1.06)
Type II	4.26 (2.35)
Type III	5.35 (2.79)
Education	
Educated at an ordinary school	21 (25.93)
Educated at an ordinary center with special sessions	39 (48.15)
Educated at a special needs education center	3 (3.70)
Home schooled	5 (6.17)
Nursery school	0.00
Not received education	10 (12.35)
Missing	3 (3.70)
Caregivers	
No. of caregivers (N)	81 (100)
Gender	
Male	7 (8.64)
Female	36 (44.44)
Missing	38 (46.92)
Age	40.29 (7.30)^a^
Working situation	
Employed	25 (30.87)
Retired or pensioner	0
Housewife/house husband	16 (19.75)
Missing	40 (49.38)
Caregiving time (daily hours)	4.05^b^ (8.22)^c^
Barthel Index, median	39.1 (22.50^)a^
Zarit-caregiver burden, median	34.53 (13.41)^a^

Source: own elaboration; ^a^ Standard deviation. ^b^ Number of daily hours reported by the person of reference in the household (mother/father). ^c^ Number of daily hours of informal caregiving conditioned to hours of informal caregiving is higher than 0

Regarding the patient’s caregivers, the weight of female relatives in this role should be noted. Caregivers in Spain spent around 8.22 h per day providing care to the children due to the limitations caused by the disease. The average Barthel Index score for patients reflected severe dependence (39), while the burden for caregivers was mild to moderate as the average Zarit Burden Interview score was 35.

The health-related quality of life of the patients and caregivers was assessed (time trade off -TTO- social tariff, as well as the VAS: Table 2). The proxy EQ-5D social tariff show a score of 0.16 (max. of 1) for patients, while the EQ-5D VAS produced a score of 54.1. For caregivers, the mean EQ-5D social tariff score was 0.49 and the mean EQ-5D VAS score was 69.1. However, when considering only patients type II, it is observed that the mean EQ-5D social tariff decreased significantly, obtaining a score equal to −0.012.

**Table 2:** Health-related quality of life (HRQOL) of patients and caregivers

Patients	All (*n* = 81)	Type II (*n* = 60)
HRQOL (TTO social tariff score)	0.158 (0.44)^a^	−0.012 (0.347)^a^
Be confined to bed	19 (23.46%)	18 (30.0%)
Unable to wash or dress by themselves	30 (37.04%)	26 (43.33%)
Unable to perform their usual activities	13 (16.05%)	11 (18.33%)
Surfer anxiety or mid depression	10 (12.35%)	5 (8.33%)
Difficulties for usual activities or selfcare	40 (49.38%)	18 (30.0%)
HRQOL (VAS score)	54.09 (26.30)^a^	53.03 (25.03)^a^
Caregivers		
HRQOL (TTO social tariff score)	0.484 (0.448)^a^	0.472 (0.475)^a^
HRQOL (VAS score)	69.1 (21.96)^a^	69.9 (20.03)^a^

^a^ Standard Deviation

The average annual cost associated with SMA reached € 33,721 (SD = 38,700) in Spain (Table 3). While 32.2% of this total cost was attributed to direct healthcare costs, which amounted to € 10,882, the average direct non-healthcare costs were € 22,839 (representing 67.7% of the total cost). Within the category of direct healthcare costs, the largest component was that of the visits to medical specialists, valued at € 7732. The next most significant direct healthcare cost was hospitalizations, estimated at € 1297. The family caregiving costs represented the largest component reaching € 21,127 (62.7% of the total cost of the illness in Spain).

**Table 3:** Average annual costs per patient (€, 2014)

Resource	Mean (SD)	% Category of cost	% Total Cost
Drugs	83 (262)	0.76%	0,25%
Medical tests	603 (721)	5.54%	1,79%
Medical visits	7732 (11,211)	71.05%	22,93%
Hospitalizations	1297 (5856)	11.92%	3,85%
GP & Emergency	244 (956)	2.24%	0,72%
Health material	920 (1183)	8.45%	2,73%
Healthcare transport	3 (13.48)	0.03%	0,01%
Direct healthcare costs	10,882 (14,974)	100.00%	32,27%
Social services	746 (2511)	3.27%	2,21%
Direct non-healthcare formal costs	746 (2511)	3.27%	2,21%
Main informal career	11,508 (19,855)	54.47%	34,13%
Other informal careers	9619 (18,666)	45.53%	28,52%
Direct non-healthcare informal costs	21,127 (30,253)	92.50%	62,65%
Other non-healthcare family costs ^a^	966 (1240)	4.23%	2,86%
Direct non-healthcare costs	22,839 (31,340)	100.00%	67,73%
TOTAL COST	33,721 (38,700)	100.00%	100,00%

^a^ Contains costs attributed to non-health transport paid by the family, as well as costs associated with housing adaptation and vehicle adaptation

When taking into consideration individuals with type II, it was observed the fact that the mean costs are higher compared with those obtained with all patients (Table 4). Concretely, mean total cost of individuals with type II was € 37,670 (€ 33,721 for all individuals). Informal care cost reached € 24,099 (€21,127 for all), formal care cost arrived at € 827 (€ 746 for all) and direct healthcare cost was € 11,580 (€ 10,882 for all individuals). The reason that explains mainly such difference in costs, especially those related to personal care (informal and formal care), might be associated with the poorer quality of life of individuals with type II.

**Table 4:** Average annual costs per patient type II (€, 2014)

Resource	Mean (SD)	% Total cost
Direct healthcare costs	11,580 (14,436)	30.74
Direct non-healthcare formal costs	827 (2700)	2.20
Direct non-healthcare informal costs	24,099 (33,240)	63.97
Direct non-healthcare costs	26,089 (34,366)	69.26
TOTAL COST	37,670 (42,579)	100.00

## Discussion

This study analyzed the social/economic burden of SMA in terms of costs and decreased HRQOL. Specifically, the average annual cost associated with SMA reaches € 33,721, of which 32.2% was attributed to direct healthcare costs and 67.8% to direct non-healthcare costs. Moreover, according to the results obtained, informal caregiving constituted a major cost component.

HRQOL is another source of information that helps define the overall societal impact of a specific health problem. The estimated average EQ-5D social tariff score for patients was 0.16. This score is significantly lower than that for young Spanish people between 16 and 20 years of age (the youngest age range available: 0.987) as estimated from the Spanish Health Survey (2011-2012), or for that of pediatric patients with Type 1 Diabetes Mellitus (0.94) [33]. Alternatively, the estimated average EQ-5D social tariff score for caregivers was 0.49 while that of the general population of the same age was 0.959 (estimated from the Spanish Health Survey, 2011-2012). These data demonstrate that patients and caregivers experience a strong deterioration in HRQOL relative to the general population. In fact, caregivers have a significantly lower quality of life than the general population after controlling for age [34], mostly due to economic factors and a lack of appropriate support. Our results reveal areas in which improvements can be made, accentuating the need for family support through social care as well as civic, patient and/or organizational support.

There is currently a lack of publicly driven research into the economic burden of SMA. One previous study on the cost of SMA in Germany estimated the average annual cost per patient to be €70,566 in 2013 [4]. Major cost drivers were proposed to be informal care cost and overall, the results were very similar to those presented here. However, there were important differences in the direct health costs that probably reflect the distinct populations studied. While we carried out a population survey, the German study was a hospital survey, which is also likely to produce an overestimation of these direct costs, in particular for type I patients that place stronger demands on hospital resources. Thus, we feel that our study provides a more representative approach to estimate e healthcare costs associated with SMA patients.

This study represents the first complete and realistic costing to date of the burden of SMA patients in Spain. The main added value of the study lies in the bottom-up approach to costing. In addition, the costs were estimated for a period of 1 year and therefore, they provide a more accurate outlook of the medium-term burden of SMA. Among rare diseases, SMA is a significant health problem with important social consequences in high-income countries. The incidence and prevalence of SMA, and its consequences in terms of mortality, morbidity, economic costs and loss of quality of life justify the attention received from health authorities and society in general. Particularly, we show that the estimated average annual cost per patient in 2014 was € 33,721 for patients in Spain. The estimated cost of SMA is higher than the social costs of other chronic diseases in Spain, such as stroke (€27,711, base year 2012) [35], symptomatic chronic heart failure (€12,995 − €18,220, base year 2012) [36] or HIV/AIDS (€17,300, base year 2010) [37]. In addition, the estimated cost of SMA is higher than the social costs of other rare diseases as ataxia (€18,776, base year 2004) [11] and similar to fragile X syndrome (€31,008, base year 2012) [38], amyotrophic lateral sclerosis (€36,194) [12] and Duchenne muscular dystrophy (€36,970, base year 2012) [39].

The findings in this study have several limitations, in particular regarding the study sample, the recruitment process and the validity of caregivers as proxy. However, other studies on rare diseases have used smaller sample sizes due to the low frequency of these diseases and the refusal rates for participation. Patients with SMA were recruited by the Spanish patient association (FUNDAME, http//www.fundame.net), and although the sample was almost evenly distributed in terms of severity or dependency, we cannot guarantee the absence of selection bias as tends to occur in studies on rare diseases. There is also a potential recall bias given that patient-based data were obtained through a questionnaire. Another potential limitation when assigning health status to children is the fact that this might be a misrepresentation. As concluded elsewhere, the values for health states when ascribed to adults are higher than when those same states are associated to children [40]. Additionally, due to the information available, the most appropriate technique to be applied in the analysis to estimate the informal care cost was proxy good method. Other alternative methods such as opportunity cost method and contingent valuation [18–20, 41] were not considered in this study.

Finally, our study was based on cross-sectional data, whereas ideally the study would be a prospective longitudinal study of a cohort of people with SMA. However, this type of study was beyond our current means. To our knowledge, no study into the cost of SMA of this nature has been carried out to date and therefore, the challenge to researchers, authorities and patient associations is to carry out such a longitudinal study in the future. It is quite frequent that cost of illness studies are criticized due to the nature of the information they provide, as well as the types of resources included and the way in which they are valued [42, 43]. Nevertheless, cost of illness studies continue to attract the particular interest of policy makers and for society as a whole [44]. Firstly, because they provide information on the real economic burden that some chronic diseases cause (not only including healthcare costs but also, non-healthcare costs), which can be useful when designing policies, programs or strategies. As shown here, the non-healthcare cost of SMA is higher than the healthcare cost (68% vs 32% respectively). Thus, the failure to take into consideration non-healthcare costs might underestimate the real impact that some chronic diseases. Furthermore, cost of illness studies complement epidemiological information and in recent years, several clinical trials on SMA have been performed and others are ongoing (see http//www.clinicaltrials.gov). In particular, advanced therapies such as intrathecal antisense oligonucleotide administration to modify splicing of the SMN2 gene [45] and intravenous gene therapy based on self complementary AAV-SMN1 [46] appear to be very promising approaches to therapy, which will surely change the epidemiological landscape of SMA types and the natural history of the disease. These advances will influence the future healthcare of SMA patients, switching to a more proactive approach as opposed to the reactive measures of complications and palliative care.

## Conclusions

In conclusion, SMA produces considerable societal costs in Spain, including very high economic costs and a deterioration in the HRQOL of the patients and caregivers. SMA represents a significant hidden cost that society should be made aware of, and that should be considered in the design, implementation and evaluation of support programs for people who suffer from this disease and their families, as well as in the economic evaluation of new treatments.

## References

[1] Pearn J. Classification of spinal muscular atrophies. Lancet. 1980;1(8174):919–22. 10.1016/s0140-6736(80)90847-8 6103267

[2] Alías L, Barceló MJ, Bernal S, Martínez-Hernández R, Also-Rallo E, Vázquez C, Santana A, Millán JM, Baiget M, Tizzano EF (2014). Improving detection and genetic counseling in carriers of spinal muscular atrophy with two copies of the SMN1 gene. Clin Genet.

[3] López-Bastida J, Oliva-Moreno J, Linertová R, Serrano-Aguilar P (2016). Social/economic costs and health-related quality of life in patients with rare diseases in Europe. Eur J Health Econ.

[4] Klug C, Schreiber-Katz O, Thiele S, Schorling E, Zowe J, Reilich P, Walter MC, Nagels KH (2016). Disease burden of spinal muscular atrophy in Germany. Orphanet J Rare Dis.

[5] von Elm E, Altman DG, Egger M (2007). The strengthening the reporting of observational studies in epidemiology (STROBE) statement: guidelines for reporting observational studies. Lancet.

[6] Munsat TL, Davies KE (1992). International SMA consortium meeting. Neuromuscul Disord.

[7] Zerres K, Rudnik-Schoneborn S (1995). Natural history in proximal spinal muscular atrophy. Clinical analysis of 445 patients and suggestions for a modification of existing classifications. Arch Neurol.

[8] Finkel RS, McDermott MP, Kaufmann P (2014). Observational study of spinal muscular atrophy type I and implications for clinical trials. Neurology.

[9] Mercuri E, Finkel R, Montes J (2016). Patterns ofdisease progression in type 2 and 3 SMA: implications for clinical trials. Neuromuscul Disord.

[10] Lopez-Bastida J, Serrano-Aguilar P, Perestelo-Perez L, Oliva-Moreno J (2006). Social-economic costs and quality of life of Alzheimer disease in the Canary Islands. Spain Neurology.

[11] Lopez-Bastida J, Monton-Alvarez F, Serrano-Aguilar P, Perestelo-Perez L (2008). Mov Disord.

[12] López-Bastida J, Perestelo-Pérez L, Montón-Alvarez F, Serrano-Aguilar P, Alfonso-Sanchez JL (2009). Social economic costs and health-related quality of life in patients with amyotrophic lateral sclerosis in Spain. Amyotroph Lateral Scler.

[13] López-Bastida J, Linertová R, Oliva-Moreno J, Posada-de-la-Paz M, Serrano-Aguilar P (2014). Social economic costs and health-related quality of life in patients with systemic sclerosis in Spain. Arthritis Care Res.

[14] Ministerio de Sanidad, Servicios Sociales e Igualdad. Costes por GRD. Available at: http://www.msssi.gob.es/estadEstudios/estadisticas/inforRecopilaciones/anaDesarrolloGDR.htm. Accessed Apr 2016.

[15] Instituto de Mayores y Servicios Sociales (IMSERSO). Ministerio de Sanidad, Servicios Sociales e Igualdad. Available at: http://www.imserso.es/imserso_01/el_inserso/index.htm. Accessed Apr 2016.

[16] Vademecum. Guía Farmacológica. 2016. Available at: https://www.vademecum.es/productos-vademecum-guia+farmacologica-3. Accessed Apr 2016.

[17] Agencia Española del Medicamentos y productos sanitarios. Available at: https://www.aemps.gob.es. Accessed Apr 2016.

[18] Hoefman RJ, van Exel J, Brouwer W (2013). How to include informal care in economic evaluations. PharmacoEconomics.

[19] van den Berg B, Spauwen P (2006). Measurement of informal care: an empirical study into the valid measurement of time spent on informal caregiving. Health Econ.

[20] van den Berg B, Brouwer W, van Exel J, Koopmanschap M, van den Bos G, Rutten F (2006). Economic valuation of informal care: lessons from the application of the opportunity costs and proxy good methods. Soc Sci Med.

[21] López-Bastida J, Linertová R, Oliva-Moreno J, Posada-de-la-Paz M, Serrano-Aguilar P, Kanavos P, Taruscio D, Schieppati A, Iskrov G, Baji P, Delgado C, von der Schulenburg JM, Persson U, Chevreul K, Fattore G; BURQOL-RD Research Network. Social/economic costs and health-related quality of life in patients with Prader-Willi syndrome in Europe. Eur J Health Econ. 2016;17(Suppl 1):99–108. 10.1007/s10198-016-0788-z 27038627

[22] Brooks R (1996). EuroQol: the current state of play. Health Policy.

[23] Drummond MF, O’Brien B, Stoddart GL, Torrance GW (1997). Methods for the economic evaluation of health care programmes.

[24] Dolan P (1997). Modeling valuations for EuroQol health states. Med Care.

[25] Stolk EA, Busschbach JJ, Vogels T (2000). Qual Life Res Int J Qual Life Asp Treat Care Rehabil.

[26] Willems DCM, Joore MA, Nieman FHM, Severens JL, Wouters EFM, Hendriks JJE (2009). Using EQ-5D in children with asthma, rheumatic disorders, diabetes, and speech/language and/or hearing disorders. Int J Technol Assess Health Care.

[27] Secnik K, Matza LS, Cottrell S, Edgell E, Tilden D, Mannix S (2005). Health state utilities for childhood attention-deficit/hyperactivity disorder based on parent preferences in the United kingdom. Med Decis Mak.

[28] Matza LS, Secnik K, Mannix S, Sallee FR (2005). Parent-proxy EQ-5D ratings of children with attention-deficit hyperactivity disorder in the US and the UK. PharmacoEconomics.

[29] Mahoney FI, Barthel DW (1965). Functional evaluation: the Barthel index. Md State Med.

[30] Shah S, Vanclay F, Cooper B (1989). Improving the sensitivity of the Barthel index for stroke rehabilitation. J Clin Epidemiol.

[31] Ruzafa CJ, Damian Moreno J (1997). Valoracion de la discapacidad fisica: el Indice de Barthel. Rev Esp Salud Pública.

[32] Hérbert R, Bravo G, Préville M. Reliability, validity, and reference values of the Zarit burden interview for assessing informal caregivers of community-dwelling older persons with dementia. Can JAging. 2000:494–507.

[33] Vázquez LA, López-Bastida J, Perez-Nieves M, Oliva-Moreno J, Villoro R, Dilla T, Merino M, Sapin H, Aranda I, Reviriego J, López-Siguero JP (2016). Impact of glycemic control and diabetic complications or comorbidities on health related quality of life in pediatric patients with type 1 diabetes mellitus and their caregivers in Spain.

[34] Oliva-Moreno J, Lopez-Bastida J, Worbes-Cerezo M, Serrano-Aguilar P (2010). Health related quality of life of Canary Islands citizens: combining a general health survey and observational studies. BMC Public Health.

[35] Alvarez-Sabín J, Quintana M, Masjuan J, Oliva-Moreno J, Mar J, Gonzalez-Rojas N, Becerra V, Torres C, Yebenes M. CONOCES investigators group. Economic impact of patients admitted to stroke units in Spain: Eur J Health Econ. 2017;18(4):449–458. 10.1007/s10198-016-0799-9 27084749

[36] Delgado JF, Oliva J, Llano M, Pascual-Figal D, Grillo JJ, Comín-Colet J, Díaz B, Martínez de La Concha L, Martí B, Peña LM (2014). Health care and non-health care costs in the treatment of patients with symptomatic chronic heart failure in Spain. Rev Esp Cardiol (Engl Ed).

[37] Trapero-Bertran M, Oliva J (2014). Economic burden of HIV/AIDS in the European context. Heal Econ Rev.

[38] Chevreul K, Gandré C, Brigham KB, López-Bastida J, Linertová R, Oliva-Moreno J, Serrano-Aguilar P, Posada-de-la-Paz M, Taruscio D, Schieppati A, Iskrov G, Gulácsi L, von der Schulenburg JM, Kanavos P, Persson U, Fattore G (2016). BURQOL-RD research network. Social/economic costs and health-related quality of life in patients with fragile X syndrome in Europe. Eur J Health Econ.

[39] Cavazza M, Kodra Y, Armeni P, De Santis M, López-Bastida J, Linertová R, Oliva-Moreno J, Serrano-Aguilar P, Posada-de-la-Paz M, Taruscio D, Schieppati A, Iskrov G, Péntek M, von der Schulenburg JM, Kanavos P, Chevreul K, Persson U, Fattore G (2016). Eur J Health Econ.

[40] Kind P, Klose K, Gusi N, Olivares PR, Greiner W (2015). Can adult weights be used to value child health states? Testing the influence of perspective in valuing EQ-5D-Y. Qual Life Res.

[41] Oliva-Moreno J, Trapero-Bertran M, Peña-Longobardo LM, del Pozo-Rubio R (2017). The valuation of informal care in cost-of-illness studies: a systematic review. PharmacoEconomics.

[42] Qian Y, McGraw S, Henne J (2015). Understanding the experiences and needs of individuals with spinal muscular atrophy and their parents: a qualitative study. BMC Neurol.

[43] Drummond M (1992). Cost-of-illness studies: a major headache?. PharmacoEconomics.

[44] Oliva J, Lopez-Bastida J, Montejo AL, Osuna R, Duque B (2009). The socioeconomic costs of mental illness in Spain. Eur J Health Econ.

[45] Chiriboga CA, Swoboda KJ, Darras BT, Iannaccone ST, Montes J, De Vivo DC, Norris DA, Bennett CF, Bishop KM (2016). Results from a phase 1 study of nusinerse (ISIS-SMN (Rx)) in children with spinal muscular atrophy. Neurology.

[46] Foust KD, Wang X, McGovern VL, Braun L, Bevan AK, Haidet AM, Le TT, Morales PR, Rich MM, Burghes AH, Kaspar BK (2010). Rescue of the spinal muscular atrophy phenotype in a mouse model by early postnatal delivery of SMN. Nat Biotechnol.

